# Defining the zerogap: cracking along the photolithographically defined Au–Cu–Au lines with sub-nanometer precision

**DOI:** 10.1515/nanoph-2022-0680

**Published:** 2023-03-09

**Authors:** Sunghwan Kim, Bamadev Das, Kang Hyeon Ji, Mahsa Haddadi Moghaddam, Cheng Chen, Jongjin Cha, Seon Namgung, Dukhyung Lee, Dai-Sik Kim

**Affiliations:** Department of Physics and Center for Atom Scale Electromagnetism, Ulsan National Institute of Science and Technology (UNIST), Ulsan 44919, Korea; and Quantum Photonics Institute, Ulsan National Institute of Science and Technology (UNIST), Ulsan 44919, Korea; Department of Physics, Chung-Ang University, Seoul, South Korea; Department of Physics, Chung-Ang University, Seoul, South Korea; Department of Physics and Astronomy, Seoul National University, Seoul 08826, Korea

**Keywords:** metal crack, photolithography, quantum conductance, sub-nanometer, zerogap fabrication

## Abstract

Cracks are formed along the photolithographically pre-determined lines with extremely high yield and repeatability, when Cu clusters are introduced between planarized Au thin films sequentially deposited on a PET substrate. These clusters act as nanometer-sized spacers preventing the formation of contiguous metallic bond between the adjacent Au layers which will render prepatterned-cracking impossible. While the effective gap width is initially zero in the optical sense from microwaves all the way to the visible, outer-bending the PET substrate allows the gap width tuning into the 100 nm range, with the stability and controllability in the ranges of 100 s and Angstrom-scale, respectively. It is anticipated that our wafer-scale prepatterned crack technology with an unprecedented mixture of macroscopic length and Angstrom-scale controllability will open-up many applications in optoelectronics, quantum photonics and photocatalysis.

## 1 Introduction

Cracks on a thin metal film on a strained flexible polymer substrate have been widely used in various applications such as mechanical strain sensors, wearable electrodes, and single molecule junctions owing to the uniform plastic deformation of the metal film [1–10]. Furthermore, since nanogaps are adapted as a sensitive platform to detect molecules in electrical and optical measurement; cracking a metal thin film is regarded as one of the feasible technologies to fabricate such structures [11–13]. Although mechanical cracks have enormous potential for sensing and other applications [14], perfectly controlling crack formation in a thin metal film remains an important challenge. For example, most studies use structurally weak points in break junctions or randomly generated cracks on a wafer, even when the end-points of cracks are lithographically defined [15]. Thereby, it is extremely difficult to draw cracks on a pre-determined, desired line with a nanometer or sub-nanometer precision, and this difficulty results in low yield of the overall devices because of the inherent randomness [16, 17].

Recently, a *zerogap* technology has been demonstrated, whereby a photolithographic, sequential deposition of planarized gold (Au) films without a spacer on a polyethylene terephthalate (PET) substrate defines pre-patterned lines, along which cracks appear by outer-bending the substrate [18]. Without the outer-bending the PET substrate, the adjacent Au planes are sufficiently well-connected to disallow any light transmission from microwaves to all the way to the visible. Thus, the gap initially considered to have a width of zero is formed along the pre-patterned lines. However, outer-bending the substrate allows the gap to open all the way to 100 nm, and the opening-closing cycle persists without any sign of fatigue for over 10,000 times. An analogy is a closable nanogap technology, whereby an alumina spacer grown by atomic layer deposition (ALD) between adjacent metals is etched out. In this case, zerogap is formed not when the substrate is at rest, but when it is inner-bent [19].

Despite many advantages of the zerogap technology, initial formation of the crack is tricky because the Au–Au interface can very often form a metallic bond which forbids cracking; the interface is completely healed and the two adjacent planes merges into one entity. Here, we greatly improve the zerogap formation yield along the photolithographic pattern, by introducing a copper (Cu) cluster layer between the Au layers at the interfaces. We reveal that the Cu layer as a spacer limits the Au–Au bonding in the sequential deposition, effectively inducing cracks at the interfaces with almost 100% yield when the sample is bent. Also, measuring the electrical conductance and terahertz (THz) transmission demonstrate stability and angstrom-scale controllability of our zerogap structure. Accordingly, it is anticipated that the mature zerogap technology will be used in optoelectronics, quantum photonics, photocatalysis, etc.

## 2 Results and discussion

### 2.1 Verification of zerogap formation

**Figure 1:**
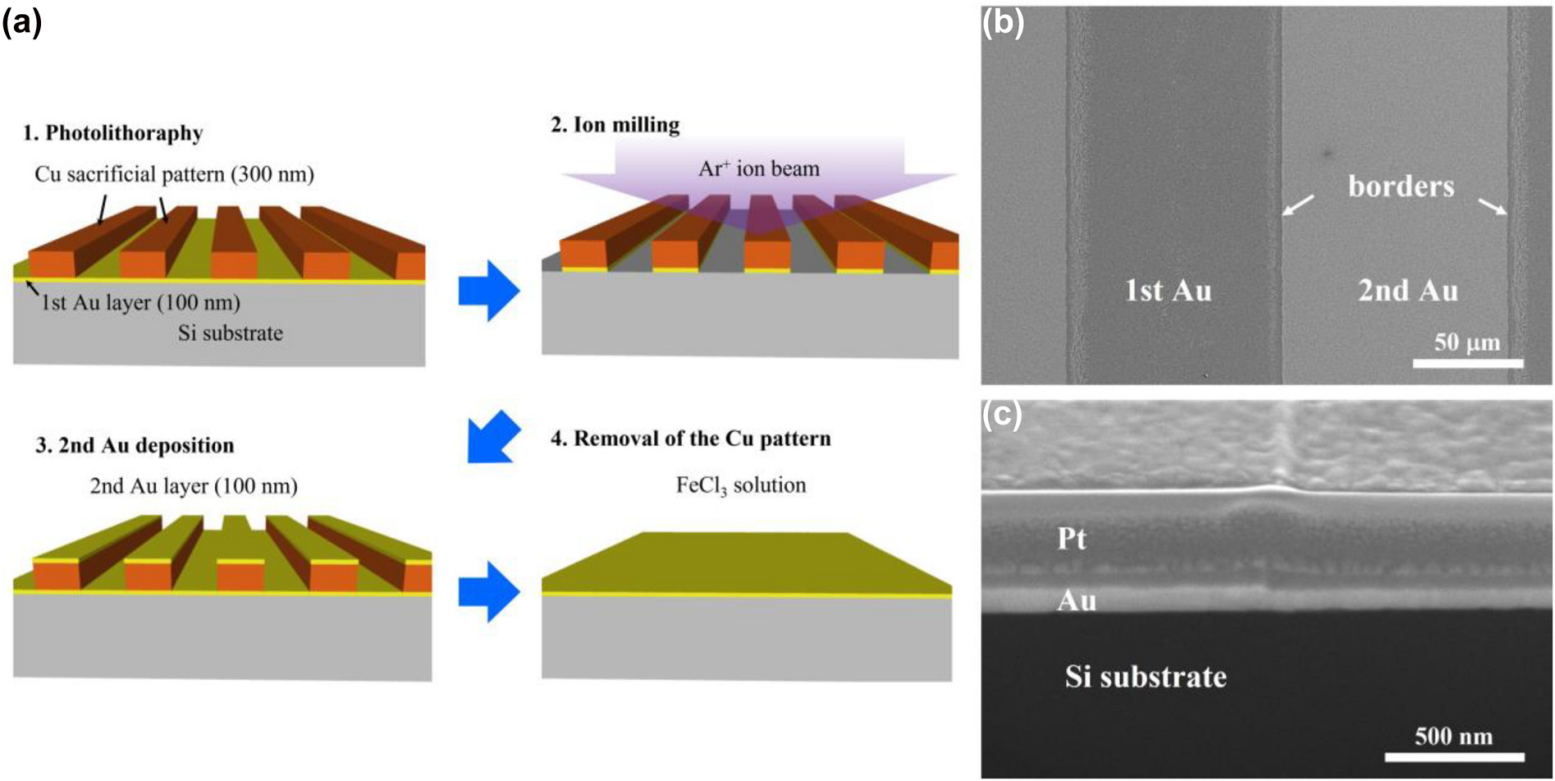
The unopenable-zerogap fabrication on a Si substrate. (a) The schematic representation of the conventional zerogap fabrication process described in the previous study [18]. (b–c) FE-SEM images after zerogap fabrication from the top (b) and the cross-section (c).

In order to examine the subtleties involved in the zerogap formation, as described in the previous study, we follow the fabrication process using a silicon (Si) substrate with 500 μm thickness. Figure 1(a). shows a schematic representation of the zerogap fabrication process which essentially follows the atomic layer lithography except for the absence of an alumina (Al_2_O_3_) spacer grown by atomic layer deposition (ALD) [20–23]. A 300 nm-thick Cu layer is used both as a hard mask in the ion milling process and a sacrificial layer in the wet-etching process. The Cu layer is firstly patterned with a stripe (1:1 line/space, 100 μm linewidth) by a standard photolithography on a 100 nm-thick Au film on the Si substrate. Then, argon (Ar) ion beam milling process is conducted to remove the Au layers unprotected by the top Cu pattern. Thereafter, the second Au layer is deposited by electron beam evaporation. Lastly, planarization is completed by chemically etching the Cu sacrificial layers with FeCl_3_ solution. The field emission-scanning electron microscope (FE-SEM) image (Figure 1(b)) shows the clear distinction between the first and second Au layers due to the sediments by the wet-etching. However, the cross-sectional view (Figure 1(c)) displays the first and second Au layers completely merged into single Au film, even though the second Au deposition is performed after a predetermined time interval. The platinum (Pt) is used only to distinguish the layers in the cross-sectional view in the FE-SEM measurements. In fact, the same procedure on the top of a PET substrate rarely yields the desired cracks even when significant strain is applied.

In atomic layer lithography or in Angstrom lithography [24–26], alumina or graphene spacers allow stable gap formation. As a solution for the effective zerogap formation with high yield, ready to be opened when strain is applied, we introduce nanometer-thin Cu spacer at the interface. From the fact that Au rarely reacts with other materials at room temperature, it is hard to alloy Au with other metals, often resulting in a bilayer stacked film with an interface [27–31]. Thus, Cu can be considered as an appropriate metal spacer since it exhibits similar characteristics as Au, with the same crystal structure and similar electrical conductivity. Additionally, titanium (Ti) or chromium (Cr), often used as an adhesive layer might be good candidates for proper spacer. Depending on how they alloy with Au, two different adhesion mechanisms are possible [32]. In case of Cr, Cr – Au alloy is formed by the Cr interdiffusion into the sublayer of Au film, whereas Ti film is stacked on the surface of Au film, to form Ti/Au bilayer partially oxidized by the substrate and the environment. Thus, Ti can act both as an adhesion layer to the substrate and as a spacer between planarized Au layers.

**Figure 2:**
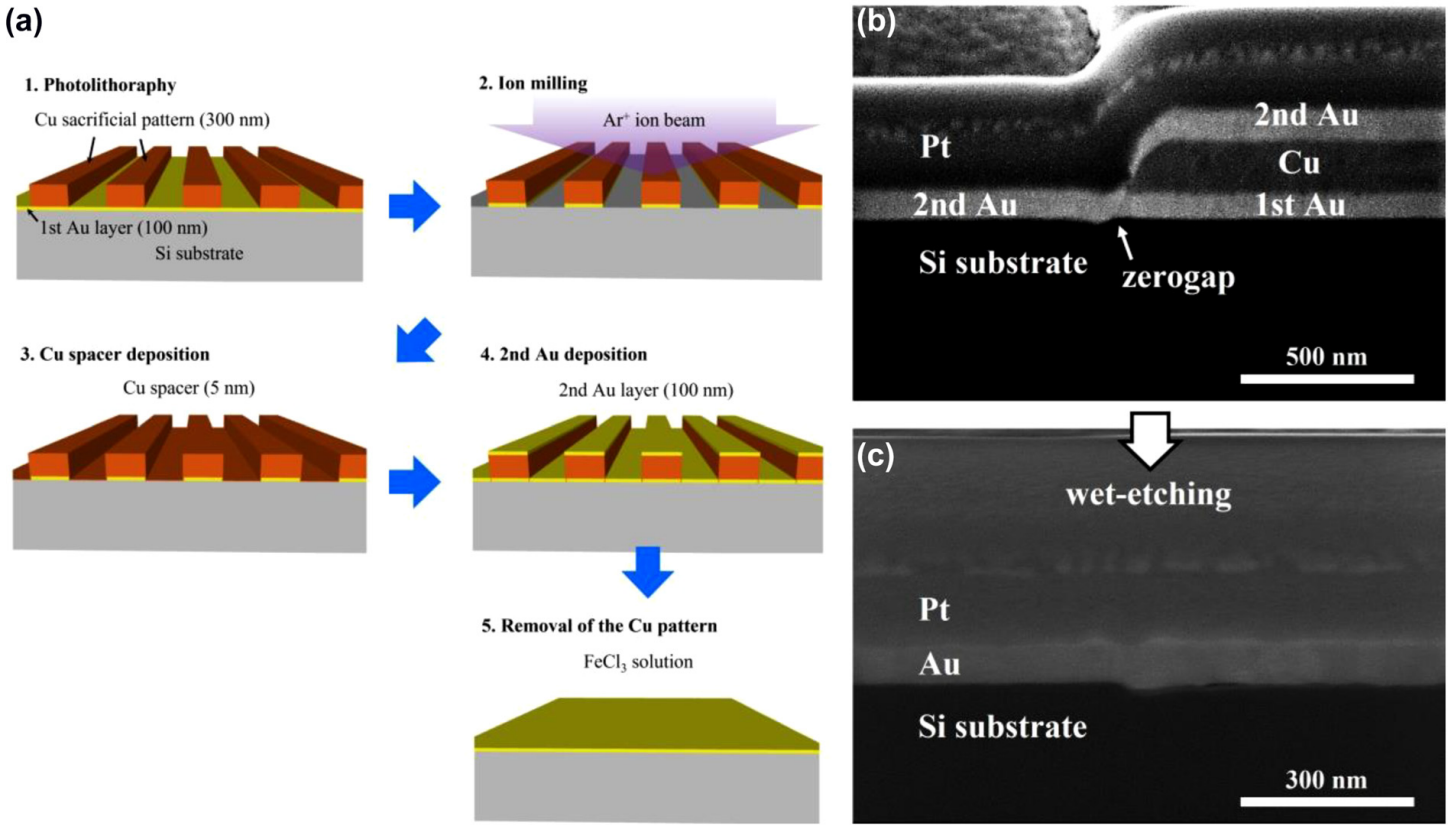
The zerogap fabrication using a Cu thin spacer. (a) The schematic representation of the modified fabrication process. (b–c) FE-SEM images before (b) and after (c) the removal of the Cu sacrificial layer. After the wet-etching process, the zerogap at the interface disappears as the Au layers bond together to form a single Au film.

**Figure 3:**
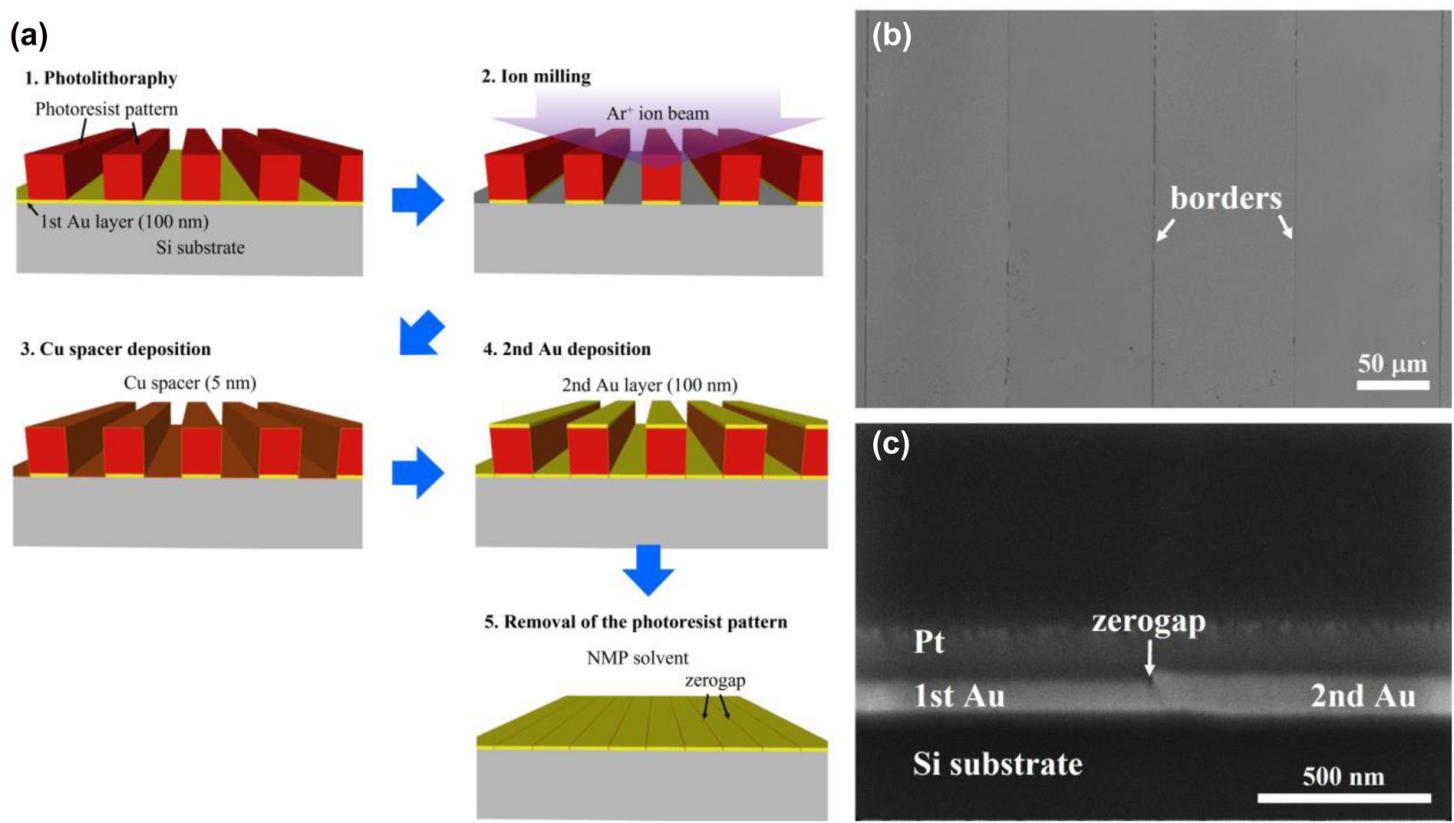
The final modified zerogap fabrication using a Cu thin spacer and a photoresist pattern as a sacrificial layer. (a) The schematic representation of the final modification process. The photolithography process is performed up to the developing step to use the photoresist pattern instead of the Cu sacrificial layer. (b–c) FE-SEM images of a zerogap completely formed on a Si substrate in the top view (b) and the cross-sectional view (c).

As the schematic representation in Figure 2(a), the additional deposition of a Cu spacer with a nominal thickness of 5 nm coats the sidewalls of the first Au layer patterned after milling. The zerogap formed at the interface is clearly observed after the second Au deposition, as shown in Figure 2(b). However, the sandwiched Cu spacer is also etched out when removing the top Cu sacrificial layer, so that zerogaps disappear again in the cross-sectional view (Figure 2(c)). Therefore, it is important to replace the etchant with an unreactive solution for the Cu spacer. To do that, an organic solvent can be used, since it always removes photoresist without changing the metal layer in the lift-off process [33]. Figure 3(a) schematically represents the final modified fabrication procedure for zerogap formation. It is noted that the Cu spacer with the nominal thickness of 5 nm is introduced before the second Au deposition. After the lift-off process using N-Methyl-2-pyrrolidone (NMP) solvent, it is confirmed that zerogap survives both in the top and cross-section FE-SEM images (Figure 3(b) and (c)). In the top view, less brightness contrast is observed compared to using the Cu sacrificial layer in Figure 1(b). In the cross-section, it is clear that the zerogap structure is finally obtained, ready to be opened by outer-bending.

### 2.2 Zerogaps on a flexible PET substrate

**Figure 4:**
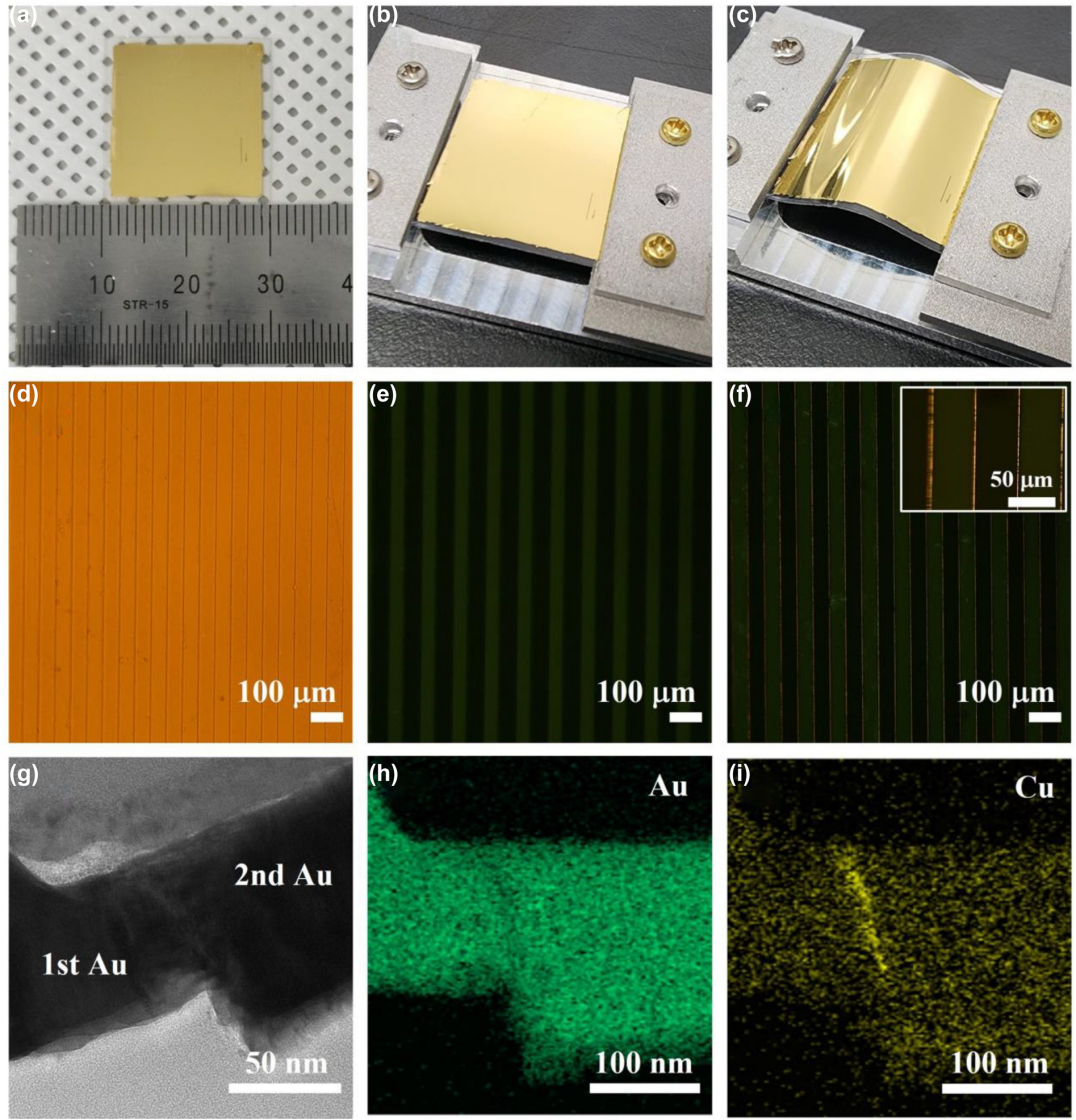
A zerogap structure on a flexible PET substrate according to the final modified fabrication. (a–c) Photographic images of a zerogap sample (a). The zerogap sample is placed on the customized strain holder at rest (b) and bent (c). (d–f) Optical microscope images of the zerogap structure observed in reflection (d) and in transmission at rest (e) and bent (f). And the inset captures the zerogap structure at 500 times magnification. (g–i) HR-TEM image of a zerogap (g), and EDX mapping analysis of the zerogap indicating the Au layers (h) and the Cu spacer at the interface (i).

Based on the procedures described in Figure 3, a zerogap structure consisting of straight lines with a 20 mm length and a 50 μm period is fabricated on a flexible PET substrate with a 250 μm thickness, as shown in Figure 4(a). And it is then placed on a custom-made strain holder consisting of a unidirectional moving slider to apply a mechanical strain to the zerogap structure. Figure 4(b) and (c) present photographic images of the zerogap sample at flat and bent, respectively. With a microscopic image on the reflection mode shown in Figure 4(d), it is found that the zerogap structure is quite uniform along the pattern. In order to confirm the zerogap structure operating under outer-bending, white light is incident from the substrate-side of an optical microscope. As can be seen in Figure 4(e), the darker stripes represent the second Au layer with the thin Cu layer buried beneath, but without any light coming through the gap because of enough electrical conductances between the first and second Au layers [34]. When the PET substrate is bent outward, light transmission is seen through the gap, indicative of wide-opening of the zerogap (Figure 4(f)). Also, the inset shows more clearly the light passing through the open gap at 500 times magnification. It is noted that the intensity of the back light is set to allow small direct transmission detection through the Au film, to clearly observe the zerogap pattern due to the contrast between the first and second Au layers enabled by the thin Cu layer below the second Au layer. It should also be noted that as the zerogap structure is bent away from the focal plane, the light from the side-gaps goes out of focus. The high resolution-transmission electron microscope (HR-TEM) measurements show a more detailed zerogap in cross-section (Figure 4(g)). In Figure 4(h) and (i), with the results of the energy dispersive X-ray (EDX) mapping analysis of Au and Cu, it is revealed that the zerogap is completely achieved by the bilayer stacked with the thin Cu spacer and the second Au layer.

**Figure 5:**
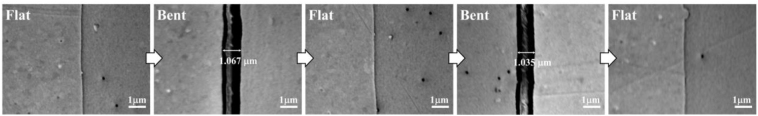
FE-SEM measurements of the zerogap structure using the tensile stage. The gap opens and closes over two cycles while the tensile stage repeatedly moves back and forth in a range of 1.5 mm.

For FE-SEM imaging, the zerogap sample is moved to a tensile stage holder built in the FE-SEM equipment. In the FE-SEM image of the flat zerogap sample shown in Figure 5, the gap is initially closed. However, with the application of an outward bending, the gap is cracked open; the stage holder moves 1.5 mm to apply the strain to the zerogap sample, and the gap width becomes ∼1 μm. Notably, the zerogap sample shows the ability of healing the interface over two cycles.

### 2.3 Applications of a zerogap structure on a flexible PET

**Figure 6:**
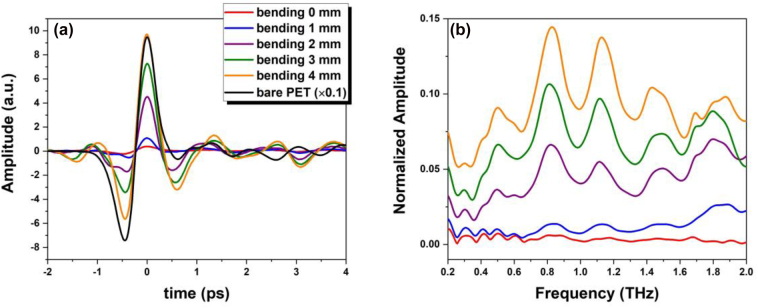
THz-TDS measurements of the zerogap structure with outer-bending of the PET substrate. THz waves transmitted through the zerogap structure in time domain (a) and in frequency domain (b).

To further investigate the optical and electrical applicability of a zerogap sample, terahertz-time domain spectroscopy (THz-TDS) and electrical measurements are performed under strains. Figure 6(a) and (b) display the THz transmission through a zerogap structure with a pattern of 50 μm period and 20 mm length on a PET substrate in the time domain and frequency domain, respectively. In the THz measurement, electromagnetic wave transmission through the gaps is recorded as the gap widens with moving the strain holder up to 4 mm. THz transmission through the zerogaps at initial state (without bending) is the same as the direct transmission through the Au film with 100 nm thickness. As the gaps are being widened, the maximum THz peak in time domain also increases proportionally, as show in Figure 6(a). The continuous tuning of gaps with high field enhancements [35] will enable a new platform for quantum plasmonics [36–40].

**Figure 7:**
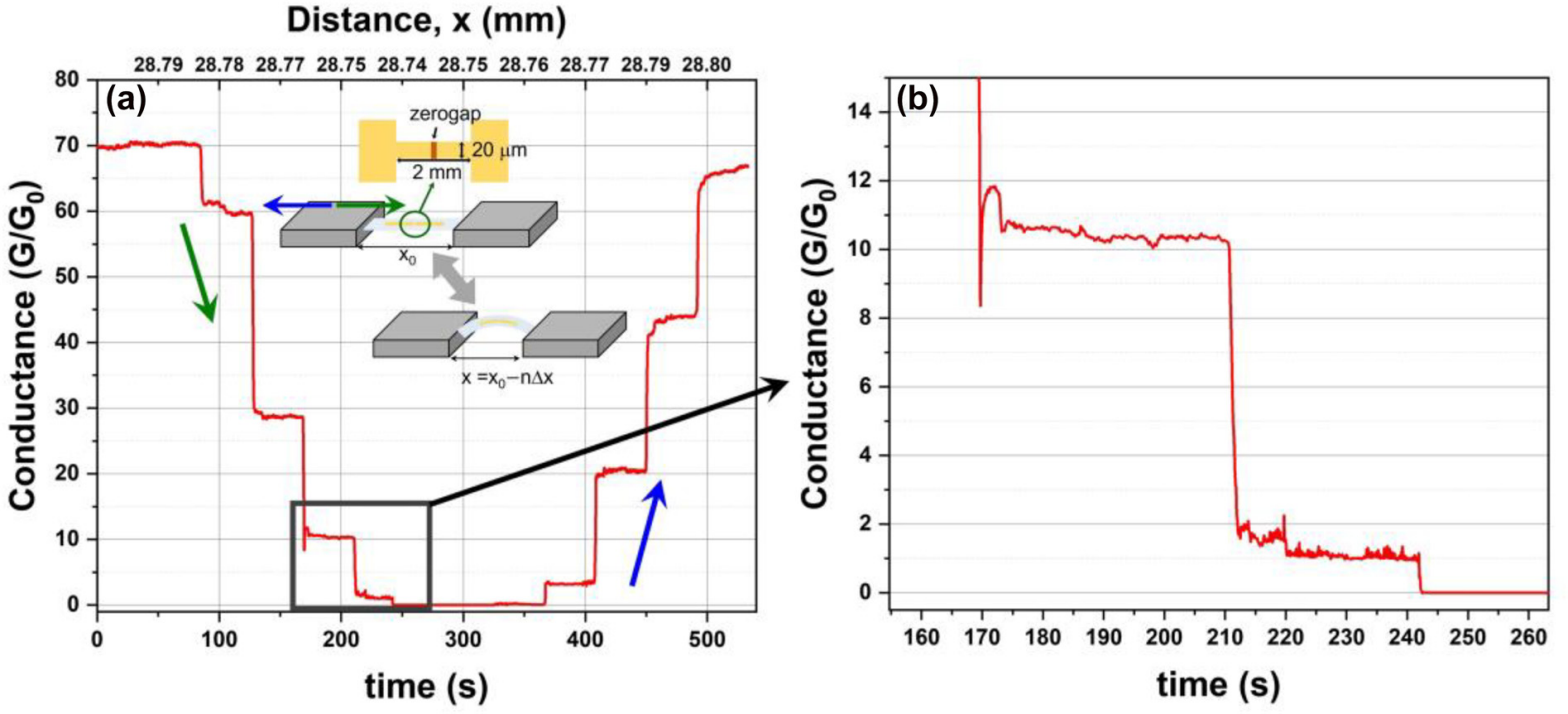
Electrical measurements using an electrode pattern with single zerogap at the center. The measurements are recorded for 30 s in one step and the stage moves 10 μm for the next step. The bending distance, *x*, is determined as the function of the step number, *n*, and the step distance, Δ*x* = 10 μm. (a) The entire measurements and (b) in the range of 4–6 steps.

Using the scheme described in Figure 5, it is easy to observe the electrical conductance with quantized channels while bending a zerogap and back. As in the inset of Figure 7(a), single zerogap is located at the center of an electrode. Then, the sample is clamped on both sides of the PET substrate. One clamp can move with a linear stage with a movement distance of 10 μm/step for the application of the outer-banding strain. To measure currents, both sides of the electrode are connected to a source meter with an applied voltage of 0.1 mV. After measuring currents for 30 s in one step, the linear stage moves to another 10 μm step to apply further strain outward. The currents start with the conductance of 70 G_0_, (G_0_ is the quantized conductance unit, 7.75 × 10^−5^ S) at a 28.80 mm μm reading (rest state). As the outer-bending radius curvature decreases, the number of Au atoms forming the current channel at the zerogap decreases, resulting in the decrease in the conductance. However, the conductance always stays around an integer with surprising stability for one measurement step (over 20 s). When moving 60 μm (6 steps), no current flows through the electrode, indicating that the gap is fully open. Shown in Figure 7(b) is a zoom around the open gap with zero conductance, achieved at slight outer-bending. At reaching 28.73 mm, the conductance becomes 1 G_0_ albeit with some high noise, but settles down at 1 G_0_ for longer than 20 s, before the next 10 μm step removes the final electrical contact. Compared to the previous study which controls quantized conductance by applying a voltage [41], it is suggested that the zerogap is effectively widened in the Angstrom-scale range by applying the strain of the PET substrate in the micron-scale range. Furthermore, considering the ambient condition of the measurement, it indicates that zerogap is very stable and controllable on Angstrom-scale comparable to the previous studies under high vacuum and vibration-free environments [42, 43]. After the conductance drops to 0, indicating that the gap completely cracked open, the stage is moved back to the original position with the same steps, reviving the quantum conductance plateaus. From the quantum conductance results, it is sufficiently demonstrated that flexible zerogap is compatible with the next generation technology such as atomic switch, resistive memory device, etc. [44, 45].

## 3 Conclusions

We investigated the zerogap formation introducing a thin metal layer such as Cu as a spacer. It is revealed that the thin metal spacer can prevent the metallic bond between the adjacent Au layers, resulting in the effective generation of zerogap at the interfaces. Based on the fabrication using metal spacer, we greatly improve the yield of zerogap structure. We demonstrated briefly the mechanical stability and reproducibility of the zerogap by the repeatable open and closed gap observation in the FE-SEM measurement. Using this structural stability and reproducibility in the THz-TDS and electrical quantum conductance measurement, zerogap sufficiently controls the gaps or cracks within Angstrom-scale resolution. As a consequence, our zerogap technique can effectively lead next-generation technologies in various applications such as optoelectronics, quantum photonics, etc.

## 4 Methods

### 4.1 Sample preparation

As per the Figure 3(a) schematics, at first, the Au film with a 100 nm thickness is deposited on a 500 μm-thick Si substrate using e-beam evaporation system (KVE-E2000, Korea Vacuum Tech). To pattern a photoresist layer, AZ5214E photoresist is coated on the Au film by a spin-coater at 4000 rpm for 60 s. I-line UV light with 365 nm wavelength, with 150 mJ/cm^2^ is irradiated to the sample located below a photomask with a stripe pattern (1:1 line/space, 100 μm linewidth) in a mask aligner (MDA-400S, MIDAS). The photoresist pattern on the Au film is obtained after immersing the sample in AZ 300 MIF developer for 50 s. Afterward, the sample is placed into the main chamber of an ion miller (KVET-IM4000, Korea Vacuum Tech). The unprotected Au layers below the photoresist pattern is milled out by argon (Ar) ion beam at an incident angle of 15° for 80 s and 80° for 60 s, sequentially (normal incidence = 0°). The nominally 5 nm-thick Cu layer spacer is deposited followed by the 100 nm-thick secondary Au layer. The second milling process is conducted in the following conditions with the Ar ion beam at the incident of 80° for 3 min to expose the sidewalls of the photoresist covered with the second Au/Cu bilayer. By immersing the sample in NMP solvent at 90 °C for 3 h to remove the photoresist layer, the zerogap structure is finally obtained. For a 250 μm-thick PET substrate, the all steps of the fabrication are the same except for a photomask with a stripe pattern of 1:1 line/space and 50 μm linewidth in the photolithography.

For the electrical measurements, a 20 mm length electrode with single zerogap at its center is prepared on a PET substrate. After completion of single zerogap fabrication on a PET substrate, photolithography is additionally implemented on it to pattern photoresist with an electrode shape of 20 μm channel width and 2 mm length in the middle, as described in the inset in Figure 7(a). The total length of the electrode pattern including both the side pads for the connection is 20 mm. Then, the sample is located on the stage of the ion miller chamber to mill out unprotected Au layers, followed by the immersion in NMP solvent. Finally, the electrode pattern with the single zerogap in the middle, ready to be opened, is obtained.

### 4.2 FE-SEM measurement

FE-SEM system (JSM-F100, Jeol) equipped with an in-built tensile stage holder is used to directly observe the gap formation with respect to different bending conditions. The tensile stage loaded with a zerogap sample is put inside the FE-SEM system. We apply the desired outward strain to the sample to crack open the gap at the pre-pattern line. At each bending, the sample is vertically moved and scanned to get the final image at the focal plane. These images give the gap formation dynamics in real time and directly showed the reproducibility of our sample.

### 4.3 THz-TDS measurement

The custom strain holder loaded with the zerogap pattern on the PET substrate is installed in the THz-TDS. In the THz-TDS, a biased GaAs THz emitter is illuminated by a mode-locked femtosecond Ti:sapphire laser operating at a 80 MHz repetition rate with a pulse duration of 130 fs at a center wavelength of 800 nm to generate THz pulse trains. THz waves generated from the emitter are collected by parabolic mirrors and are focused on the zerogap sample at normal incidence. The transmitted THz pulse is collected again by parabolic mirrors and detected via electro-optic sampling with a ZnTe crystal with (110) orientation. Time traces of the THz waves are recorded by moving the delay stage of the pump pulses.

### 4.4 Electrical measurement

The electrode sample with the single zerogap at its center as described in the Section 4.1 is placed on a clamp holder consisting of a linear moving stage. Both sides of the PET substrate are clamped to suspend the sample. Both electrode side pads are then connected to a source meter unit (Keithley 2450, Tektronix) to measure currents by applying constant voltage of 0.1 mV. The linear moving stage shifts 10 μm per step to apply strain on the zerogap bent outwardly. At each step, the currents are recorded for 30 s. After 6 steps of outer-bending, the linear stage creeps back to the original position by another 6 steps in reverse.
